# Multi-Omics Reveals Gut Microbiota Shifts and Hepatic Metabolic–Immune Alterations in “Short-Leg” Malformed Frog (*Pelophylax nigromaculatus*)

**DOI:** 10.3390/ani16132069

**Published:** 2026-07-04

**Authors:** Dan Zeng, Qin Qin, Ming Yang, Zi’ao Wang, Jianguo Xiang, Xiaoqing Wang, Yazhou Hu

**Affiliations:** 1 College of Life and Environmental Sciences, Hunan University of Arts and Science, Changde 415000, China; zengdan2013@163.com; 2 Fisheries College, Hunan Agricultural University, Changsha 410128, China; scxy@hunau.edu.cn (Q.Q.); jgxianghau@163.com (J.X.); wangxiao8258@126.com (X.W.); 3 Jiangsu Coast Development Group Co., Ltd., Nanjing 210000, China; 4 College of Animal Science and Technology, Hunan Biological and Electromechanical Polytechnic, Changsha 410127, China; 13739069939@163.com

**Keywords:** *Pelophylax nigromaculatus*, malformation syndrome, gut microbiota, hepatic transcriptome, immune–metabolic disruption

## Abstract

Amphibian malformation syndromes significantly impact both conservation efforts and aquaculture, yet their underlying systemic pathophysiological mechanisms remain poorly characterized. This study comprehensively examines the multi-level pathological processes associated with the “short-leg” malformation syndrome in the black-spotted frog (*Pelophylax nigromaculatus*) using an integrated methodology, encompassing morphological, histopathological, gut microbiome, and hepatic transcriptomic analyses. Affected frogs demonstrated shortened limbs, impaired motor function, and a distinctive metabolic phenotype, including increased body weight despite a shorter body length, accumulation of visceral fat, and shortened intestines. Gut microbiota analysis identified significant compositional shifts, characterized by a decreased Firmicutes-to-Bacteroidota ratio, expansion of pro-inflammatory Proteobacteria, and reduction in beneficial Actinobacteriota, suggesting microbial niche restructuring that likely promotes metabolic and inflammatory disorders. Hepatic transcriptome profiling revealed 2617 differentially expressed genes, demonstrating a clear molecular dichotomy with concurrent up-regulation of immune-related pathways (e.g., neutrophil extracellular trap formation, complement cascades, and inflammatory signaling) and broad suppression of metabolic pathways (e.g., lipid oxidation, nutrient absorption, and PPAR and renin–angiotensin systems). This integrated analysis illustrates that the malformation syndrome represents a systemic pathophysiological state involving dysfunction of the gut–liver axis, characterized by the coexistence of gut microbiota alterations, hepatic metabolic suppression, and immune activation. These findings provide a framework for understanding amphibian malformations and suggest potential strategies to improve health outcomes in aquaculture.

## Simple Summary

The black-spotted frog, *Pelophylax nigromaculatus*, is an economically valuable species in East Asian aquaculture, yet a “short-leg” malformation syndrome has emerged as a severe disorder that leads to death and reduces farming productivity. In this study, black-spotted frogs with a “short-leg” malformation were investigated to understand what goes wrong inside their bodies. It was found that affected frogs are not just deformed externally; they also show unusual internal changes, including increased body fat, a shorter intestine, and an altered gut bacterial community, with more harmful bacteria and fewer beneficial ones. At the liver level, genes related to the immune system were overactive, while those responsible for normal metabolism were suppressed. This suggests that the malformation is not a simple local defect but a whole-body disease involving concurrent abnormalities in both the gut and liver. By understanding these hidden connections, malformation syndromes can be better diagnosed, prevented, or treated in amphibian aquaculture and conservation programs.

## 1. Introduction

The black-spotted frog (*Pelophylax nigromaculatus*) is widely distributed across East Asia, including central, northern, and northeastern China; the Russian Far East; the Korean Peninsula; and Japan [1]. Owing to substantial market demand in China, this species holds considerable economic value, with an annual output approaching 100,000 metric tons. However, commercial cultivation of *P. nigromaculatus* encounters considerable difficulties resulting from the species’ high susceptibility to diseases, frequently leading to severe economic losses. Recent outbreaks of pathogenic and metabolic disorders, such as metamorphosis syndrome, red-leg disease, and meningitis-like disease [2], have resulted in developmental abnormalities in juveniles, reduced survival rates, and stunted adult growth, thereby compromising production efficiency. Previous studies have implicated poor water quality [3], environmental pollutants [4,5], nutritional imbalances, and pathogen transmission under high-density farming conditions [6] as major contributing factors. Therefore, understanding the pathogenic mechanisms underlying major diseases and developing eco-friendly management strategies are essential for sustainable aquaculture.

Developmental malformation syndrome represents a particularly severe disorder characterized by impaired metamorphosis or post-metamorphic defects. Affected individuals exhibit limb deformities, spinal abnormalities (e.g., scoliosis), visceral malformations, and functional impairments that substantially reduce viability [7,8]. These malformations typically occur when environmental stressors, including pollutants, parasites, nutritional deficiencies, and physical injuries, interfere with normal anatomical development during the larval phase [9]. Despite the recognition of these factors, the precise pathophysiological links between environmental stressors and specific malformation phenotypes remain inadequately defined, particularly within farmed amphibian populations. The gut–liver axis represents a bidirectional communication pathway in which gut microbiota-derived metabolites enter the portal circulation and modulate hepatic metabolic and immune functions. Disruption of gut microbial homeostasis can lead to increased intestinal permeability, facilitating the translocation of bacterial products to the liver, where they trigger inflammatory responses and metabolic reprogramming. Although the gut–liver axis has been extensively studied in mammals, its role in amphibian pathophysiology remains largely unexplored [10]. Recent studies have started adopting integrative methods, linking gut microbiota composition to hepatic metabolic pathways to elucidate disease mechanisms in amphibians [11], or exploring the gut–liver axis’s role in amphibian health [12]. These emerging studies emphasize the importance of transitioning from single-factor approaches towards comprehensive, multi-dimensional frameworks connecting gut microbiota, host metabolism, and tissue pathology.

Through long-term aquaculture observations, a distinct “short-leg” malformation syndrome was identified in juvenile *P. nigromaculatus* post-metamorphosis. Although tail resorption occurred normally in malformed frogs, these individuals demonstrated significantly decreased feeding behaviors, ultimately resulting in death and severely impacting farming productivity. To clarify the underlying mechanisms of this syndrome, an integrated investigation was conducted, involving morphological analyses, histopathological examinations, gut microbiota characterization, and hepatic transcriptomic sequencing. By integrating multiple analytical levels, bridging external phenotypes, tissue pathology, microbial ecology, and host molecular responses, this study aims to provide novel insights into the etiology of limb deformities in *P. nigromaculatus* and inform effective aquaculture mitigation strategies.

## 2. Materials and Methods

### 2.1. Sample Collection

Adult *P. nigromaculatus* were categorized by external morphology into a normally developed control group (CON, *n* = 30) and a group exhibiting the characteristic “short-legged” malformation (MAL, *n* = 25). Comprehensive morphometric and functional data were collected, including body weight (BW), snout-to-vent length (SVL), forelimb length (FL), hindlimb length (HL), and leaping distance (LD). The frog was placed on a flat, non-slip surface. After a gentle tail touch stimulus, the maximum distance of a single forward leap was recorded. Each frog was tested three times, with at least 5 min of rest between trials, and the average value was used.

Following measurements, dissections were performed to obtain organ-specific metrics such as liver weight (LW), fat mass (FM), and intestinal length (IL). All data are presented as mean ± SD. For most morphometric parameters (BW, SVL, LD, LW, FM, and IL), differences between the CON and MAL groups were evaluated using independent-samples *t*-tests, with *p* < 0.05 considered statistically significant and *p* < 0.01 considered highly significant. For forelimb and hindlimb lengths, an analysis of covariance (ANCOVA) was performed with SVL as a covariate to correct for body size differences. Assumptions of homogeneity of regression slopes and normality were verified.

### 2.2. Histopathological Analysis

Following dissection, key tissues (liver and intestine) were collected immediately from representative individuals in both groups. For histological analysis, liver tissues from three frogs per group (CON and MAL) were used. Samples were fixed in 4% paraformaldehyde (PFA) solution for 24–48 h at 4 °C to preserve cellular integrity. Fixed tissues were dehydrated using a graded ethanol series, cleared in xylene, and embedded in paraffin wax. Serial sections (5 μm thick) were cut using a rotary microtome, mounted on glass slides, deparaffinized, rehydrated, and stained using standard hematoxylin and eosin (H&E) protocols. The slides were then coverslipped using neutral balsam. All slides were examined and imaged using a light microscope equipped with a digital camera.

### 2.3. Gut Microbiota Analyses

Intestinal contents were collected from three normal and short-limbed malformed *P. nigromaculatus* (*n* = 3 per group). These contents were collected from each frog individually. Total microbial DNA was extracted using an E.Z.N.A.^®^ DNA Kit (Omega Bio-Tek, Norcross, GA, USA). The V3-V4 region of the 16S rRNA gene was amplified using primers 338F (5′-ACTCCTACGGGAGGCAGCAG-3′)/806R (5′-GGACTACHVGGGTWTCTAAT-3′) and sequenced on the Illumina Nextseq2000 platform (Illumina, San Diego, CA, USA) using a paired-end 2 × 250 bp configuration. Raw FASTQ reads were demultiplexed, quality-filtered using fastp v0.19.6 (sliding window of 50 bp with average quality ≥ 20; reads < 50 bp after trimming discarded), and merged using FLASH v1.2.7 (minimum overlap of 10 bp and maximum mismatch ratio of 0.2). Samples were distinguished by exact barcode matching and up to 2 primer mismatches. Optimized sequences were clustered into operational taxonomic units (OTUs) at 97% similarity using UPARSE v 7.1, and they were taxonomically annotated against the SILVA (v138) database. All samples were rarefied to 20,000 sequences before further analysis. To identify microbial taxa distinguishing between groups, partial least squares discriminant analysis (PLS-DA) and linear discriminant analysis effect size (LEfSe) were performed. Differentially abundant taxa were identified with thresholds of an LDA score > 2.0 and *p* < 0.05.

### 2.4. Sampling, RNA Extraction, and Illumina Sequencing

Liver samples were collected individually from each frog. Total RNA was extracted from the liver tissues of three normal and short-limbed malformed *P. nigromaculatus* (*n* = 3 per group) using QIAzol Lysis Reagent (Qiagen, Hilden, Germany). RNA quality was assessed using a NanoDrop2000 spectrophotometer (Thermo Fisher Scientific, Waltham, MA, USA) and an Agilent 5300 Bioanalyzer (Agilent Technologies, Santa Clara, CA, USA); only samples with an RNA Integrity Number (RIN) ≥ 7.0 were used for library construction. High-quality RNA samples were selected for library construction. Strand-specific mRNA libraries were prepared using an Illumina^®^ Stranded mRNA Prep Kit (Illumina, San Diego, CA, USA) and sequenced on the NovaSeq X Plus platform (PE150) (Illumina, San Diego, CA, USA).

Raw sequencing data were processed with fastp to remove adapters and low-quality reads. De novo transcriptome assembly was performed using Trinity, and transcripts were clustered into non-redundant unigenes using CD-HIT. Assembly quality was evaluated using TransRate and BUSCO. Functional annotation was obtained by aligning unigenes against the NR, Swiss-Prot, KEGG, GO, and eggNOG databases using Diamond with an E-value cutoff of 1 × 10^−5^.

### 2.5. Gene Expression Quantification and Differential Expression Analysis

Gene expression levels were quantified using RSEM (http://deweylab.github.io/RSEM/ (accessed on 27 June 2026)). Differential expression analysis was conducted using DESeq2 (https://bioconductor.org/packages/release/bioc/html/DESeq2.html (accessed on 27 June 2026)), with thresholds set at |log_2_FC| > 1 and FDR < 0.05. Gene Ontology (GO) and KEGG pathway enrichment analyses of differentially expressed genes (DEGs) were performed using Goatools v1.2.3 and the Python scipy package v1.10.1, respectively. *p*-values were corrected for multiple comparisons using the Benjamini–Hochberg false discovery rate (FDR) procedure, and adjusted *p* < 0.05 was considered statistically significant. All bioinformatic analyses were completed on the Majorbio Cloud Platform (https://cloud.majorbio.com (accessed on 27 June 2026)).

### 2.6. Validation of Transcriptome Data via qPCR

To validate the transcriptomic results, quantitative real-time PCR (qPCR) was performed on selected DEGs. The total RNA previously used for transcriptome sequencing was reverse-transcribed into cDNA using a PrimeScript™ RT Reagent Kit (Takara Bio Inc., Kusatsu, Shiga, Japan). Gene-specific primers were designed with Primer Premier 6.0 (Premier Biosoft, San Francisco, CA, USA) and synthesized by Tsingke Biotechnology (Beijing, China) (Table S1). qPCR reactions were performed in triplicate using a LightCycler^®^ 480 System (Roche Diagnostics, Basel, Switzerland) and 2 × Universal SYBR qPCR Master Mix (YUNBIO Co., Ltd., Hunan, China, Cat. No. qPF001). The reaction conditions included an initial denaturation at 95 °C for 30 s, followed by 40 cycles of 95 °C for 15 s, 58 °C for 15 s, and extension at 72 °C for 15 s. The *18S* rRNA gene was used as an internal reference for normalization. Relative gene expression levels were calculated using the 2^−ΔΔCt^ method.

## 3. Results

### 3.1. Comprehensive Morphological, Functional, and Anatomical Alterations in Malformed Frogs

Based on morphological comparisons, pronounced differences were observed between the control (CON) and malformed (MAL) groups across multiple traits (Figure 1). In terms of body size and coloration, CON frogs exhibited normal body proportions with vibrant green dorsal skin bearing characteristic dark stripes, and the skin appeared smooth and lustrous. In contrast, MAL frogs were notably smaller, with dull coloration ranging from dark green to grayish-green, indistinct or absent stripes, and a rough skin texture. Regarding limb development, CON frogs displayed well-proportioned limbs with elongated hindlimbs and distinct joints. The MAL group exhibited a pronounced “short-leg” phenotype, with hindlimbs dramatically shortened relative to body size, underdevelopment of the tibiofibular segment, and abnormal joint curvature. Upon dissection, marked differences in visceral features were observed between the two groups. CON frogs exhibited normally positioned viscera with clear organ boundaries and no apparent abnormalities. In contrast, MAL frogs displayed conspicuous dark red or brownish visceral masses, with obscured organ contours and evidence of visceral fat accumulation. These morphological features collectively indicate that the malformation syndrome encompasses not only pronounced limb defects but also systemic alterations in internal organ structure and body condition.

Consistent with this phenotype, the MAL group exhibited significantly reduced body measurements. SVL was significantly shorter in malformed frogs than in controls (Figure 2A; t_(53)_ = 2.601, *p* = 0.012). Similarly, FL and HL were also significantly reduced in the MAL group (Figure 2B, C; FL: F_(1,52)_ = 8.071, *p* = 0.006; HL: F_(1,52)_ = 16.173, *p* < 001). This limb shortening resulted in notable functional impairment, as the LD was significantly reduced in the MAL group (Figure 2D; t_(8)_ = 9.264, *p* < 001). Notably, despite their smaller size and shorter limbs, the MAL group exhibited a significantly greater body weight (BW) than the control group (Figure 2E; t_(83)_ = −2.170, *p* = 0.035). Dissections revealed substantial internal organ changes in malformed frogs. Specifically, both LW and FM were significantly increased in the MAL group compared to the control group (Figure 2F, G; LW: t_(49)_ = −2.332, *p* = 0.024; FM: t_(45)_ = −2.880, *p* = 0.006). Additionally, IL was significantly shorter in malformed individuals (Figure 2H; t_(29)_ = 2.813, *p* = 0.009). These findings suggest that the malformation syndrome involves extensive internal anatomical and metabolic disruptions in addition to external limb defects.

Normalization of physiological indices to body size revealed multisystem physiological dysregulation in malformed *P. nigromaculatus* (Table S2). Compared to controls, malformed frogs had significantly higher hepatosomatic indices (MAL: 0.126 ± 0.103 vs. CON: 0.071 ± 0.048, t_(49)_ = −2.535, *p* = 0.015) and increased lipid-to-body weight ratios (MAL: 0.033 ± 0.015 vs. CON: 0.017 ± 0.008, t_(45)_ = −4.846, *p* < 001), indicating severe hepatic enlargement and abnormal fat accumulation. Conversely, malformed frogs displayed significantly reduced intestinal ratios (MAL: 1.433 ± 0.292 vs. CON: 1.716 ± 0.371, t_(29)_ = 2.318, *p* = 0.028), confirming intestinal shortening independent of body size. Collectively, these body size-normalized parameters demonstrate that the malformation syndrome involves extensive physiological disruptions beyond mere growth impairment, including hepatomegaly, excessive fat deposition, and shortened intestines.

### 3.2. Histopathological Analysis Reveals Concurrent Intestinal and Hepatic Injuries

Histopathological examination (H&E staining) revealed distinct morphological abnormalities in the intestinal and hepatic tissues of malformed frogs. In the intestine, malformed frogs exhibited thickened, densely packed, and partially fused intestinal villi, along with increased inflammatory cell infiltration in the lamina propria and hyperchromatic epithelial cell nuclei indicative of cellular stress (Figure 3A,B). In the liver, the normal lobular architecture was severely disrupted, and hepatocytes were arranged irregularly. The hepatocyte cytoplasm contained numerous vacuoles of variable size, suggestive of fatty degeneration, accompanied by dilated hepatic sinusoids, substantial inflammatory infiltration, and pyknotic nuclei (Figure 3C,D). These histological observations collectively demonstrate structural disruptions and tissue-level injuries in both the intestinal and hepatic tissues of malformed frogs.

### 3.3. Compositional Shifts in the Gut Microbiota

An analysis of 16S rRNA gene sequencing revealed substantial changes in the diversity and composition of gut microbiota in malformed *P. nigromaculatus*. The Venn diagram in Figure 4A shows that the CON group possessed 1194 operational taxonomic units (OTUs), whereas the MAL group had only 722 OTUs, with 427 OTUs shared between the two groups. PLS-DA at the phylum level demonstrated clear separation between the CON and MAL groups, explaining approximately 47% of the total variance, with notable intra-group consistency and inter-group distinction (Figure 4B). Figure 4C displays the top 20 intestinal microbial phyla by relative abundance. The Firmicutes/Bacteroidota (F/B) ratio was calculated from the mean relative abundances. The F/B ratio was 7.33 in the control group and 1.96 in the malformed group, indicating a marked decrease in malformed frogs. While a core microbiome was present in both groups, malformed frogs exhibited decreased Firmicutes abundance and increased levels of Proteobacteria, Fusobacteriota, and Bacteroidota compared to controls. LEfSe analysis (LDA score > 2.0) identified specific microbial taxa associated with malformation syndrome (Figure 4D). The cladogram illustrated distinct microbial profiles between the groups: The MAL group was characterized by enriched Firmicutes-affiliated taxa, including *Peptostreptococcaceae*, *Tannerellaceae*, *Macellibacteroides*, *Pygmaiobacter*, *Sporacetigenium*, and *Terrisporobacter*, clustering tightly on the phylogenetic tree. In contrast, the CON group was characterized by Actinobacteriota and related lineages such as *Actinobacteria*, *Solirubrobacteraceae*, *Conexibacter*, *Microvirga*, and *Solirubrobacter*, forming a separate cluster. Minimal phylogenetic overlap existed between characteristic taxa of the two groups, suggesting that malformation syndrome induces systemic restructuring of the gut microbial community rather than altering only individual taxa abundances.

### 3.4. RNA-Seq of Liver and Quality Assessment

To clarify the intrinsic regulatory mechanisms underlying the observed metabolic disturbances and morphological malformations, comparative transcriptomic analysis of the liver tissues was conducted. RNA sequencing of six libraries yielded 19–23 million clean reads per sample. All libraries showed high-quality metrics, including Q20 scores >95.74%, uniform GC content (~44%), and an average uniquely mapping rate of 84% (Table 1).

### 3.5. De Novo Assembly and Functional Annotation of the Liver Transcriptome

High-quality clean reads from all samples were assembled de novo into 96,814 transcripts, which were clustered further into 67,985 non-redundant unigenes. Length distribution analysis indicated that 49% (33,545) of the unigenes were 200–500 bp. Additionally, 18% (12,439) exceeded 1500 bp, indicating good assembly continuity with an N50 value of 1681 bp (Figure S1A).

Functional annotation of unigenes was performed by querying multiple public databases. Overall, 48.6% (33,011) of the total unigenes were annotated in at least one database. The most comprehensive annotations were from the NR database (47.7%, 32,447 unigenes), followed by the eggNOG (33.1%), Swiss-Prot (29.9%), KEGG (29.2%), and GO (28.4%) databases (Figure S1B). This robust annotation profile provides a reliable foundation for subsequent functional and pathway analyses.

### 3.6. Global Hepatic Transcriptomic Alterations in Malformed Frogs

Transcriptomic profiling revealed significant differences in hepatic gene expression between malformed and control frogs. A Venn diagram indicated 21,924 genes expressed commonly in both groups, with 14,392 genes unique to CON and 6119 genes unique to MAL, indicating substantial transcriptomic divergence (Figure 5A). Differential expression analysis identified 2617 DEGs (|log_2_FC| > 1, FDR < 0.05) (Figure 5B and Table S3). Gene expression changes were notably asymmetrical, with 1624 genes significantly down-regulated and 993 genes up-regulated in malformed frogs. Hierarchical clustering based on these DEGs clearly segregated samples into two distinct clusters matching their phenotypic groups (Figure 5C).

### 3.7. Functional and Pathway Enrichment Analyses of DEGs

GO enrichment analysis identified 21 significantly enriched terms (padjust < 0.05) among the DEGs, spanning the three main categories: Cellular Component (CC), Biological Process (BP), and Molecular Function (MF) (Figure 6 and Table S4). Notably, the most significantly enriched terms were predominantly related to cellular structures and metabolic processes. In the CC category, DEGs were significantly enriched in “extracellular region”, “membrane”, and other structural terms such as “cellular anatomical entity”, suggesting substantial disruptions to the cellular architecture and microenvironment. Within the BP category, prominent enrichments were related to the catabolism of metabolites, including “carboxylic acid catabolic process”, “organic acid catabolic process”, and specifically “amino acid catabolic process”, indicating significant reprogramming of energy metabolism. In the MF category, DEGs showed significant enrichment in binding and catalytic functions, including “tetrapyrrole binding”, “heme binding”, and “peptidase activity”. These functional profiles imply that the malformation syndrome in frogs is closely associated with disruptions in fundamental cellular structures and heightened catabolic metabolism.

To further dissect transcriptional dysregulation, we performed KEGG pathway enrichment analysis separately for up- and down-regulated DEGs (Table S5). This analysis highlighted distinct pathway alterations. Up-regulated DEGs predominantly enriched immune, inflammatory, and protein synthesis pathways. The most significantly enriched pathways included “Ribosome” (27 genes, padjust = 1.01 × 10^−7^), “Coronavirus disease–COVID-19” (38 genes, padjust = 1.12 × 10^−6^), “Neutrophil extracellular trap formation” (23 genes, padjust = 1.50 × 10^−6^), and “Complement and coagulation cascades” (16 genes, padjust = 3.20 × 10^−4^), reflecting active immune responses and potential tissue injury. Additionally, several amino acid metabolism pathways (Glycine, serine, and threonine metabolism, and Arginine and proline metabolism) were significantly up-regulated (Figure 7A). In contrast, down-regulated DEGs were predominantly associated with impaired nutrient absorption and metabolism. The most significantly suppressed pathways included “Protein digestion and absorption” (17 genes, padjust = 0.003), “Mineral absorption” (12 genes, padjust = 0.004), and “Vitamin digestion and absorption” (11 genes, padjust = 0.007). Important lipid metabolism pathways, such as “Linoleic acid metabolism” (7 genes, padjust = 0.016) and “Ether lipid metabolism” (9 genes, padjust = 0.019), were also significantly down-regulated (Figure 7B).

Within the PPAR signaling pathway, genes critical for lipid metabolism, including *K00029 (meaB)*, *K08750 (FABP1)*, *K06259 (CD36)*, *K01897 (ACSL1)*, and *K19523 (CPT1B)*, as well as *K06086 (SORBS1)* involved in adaptive thermogenesis, were significantly down-regulated in the malformed group (Table 2 and Figure S2A). Conversely, genes related to ubiquitination (*K08770*, *UBC*) and gluconeogenesis (*K01596*, *E4.1.1.32*) were significantly up-regulated. Furthermore, all detected DEGs in the renin–angiotensin system, including *K01283 (ACE)*, *K09708 (ACE2)*, *K08780 (CPA3)*, *K04166 (AGTR1)*, *K11141 (ENPEP)*, and *K11140 (ANPEP)*, were down-regulated (Table 2 and Figure S2B). To verify the reliability of the RNA-seq results, we conducted qPCR analysis on 12 selected DEGs across two signaling pathways. Expression patterns from the qPCR and RNA-seq analyses were strongly concordant, confirming the directional expression trends (Figure 8). This correlation supports the accuracy and reliability of the RNA-seq data.

## 4. Discussion

The developmental malformation syndrome in amphibians involves complex processes with multifactorial etiologies. Through integrated morphological, gut microbiome, and hepatic transcriptomic analyses, this study systematically uncovers the intricate pathophysiological network underlying the “short-leg” malformation syndrome in *P. nigromaculatus*. Our findings demonstrate that this malformation is not merely a simple limb defect but rather a systemic disorder involving metabolic disturbances, gut microbiota alterations, and disruptions in key hepatic immune and metabolic pathways.

### 4.1. Malformation Syndrome Presents Comprehensive Morphological and Metabolic Abnormalities

Malformed frogs exhibited the typical “short-leg” phenotype, characterized by a markedly reduced body size and severely impaired locomotion, a combination detrimental to survival [13]. Beyond these external deformities, malformed individuals also displayed prominent internal abnormalities, including darkened visceral tissues with indistinct organ boundaries and evidence of visceral lipid accumulation. Importantly, our results indicate that this syndrome represents a systemic metabolic disorder rather than a localized developmental defect. This conclusion is supported by the paradoxical increase in body weight despite a significantly reduced SVL, FL, and HL, indicating severe disruption of energy homeostasis. The elevated hepatosomatic index and lipid-to-body weight ratio provide clear, size-adjusted evidence of hepatomegaly and obesity. This disproportionate expansion of metabolically active tissues is consistent with excessive lipid accumulation and early hepatic steatosis. Hepatic steatosis is a well-recognized stress-related metabolic disturbance in amphibians [14,15]. Furthermore, the reduced intestinal ratio confirms that intestinal shortening is an intrinsic and size-independent characteristic of the syndrome. This structural alteration likely impairs nutrient absorption capacity [16]. Thus, impaired nutrient assimilation coupled with abnormal lipid deposition constitutes the core of this metabolic imbalance.

### 4.2. Histopathological Evidence of Gut–Liver Axis Dysfunction

Histopathological examination revealed simultaneous structural damage in both the intestine and liver of malformed frogs, providing direct morphological evidence of systemic tissue injury. In the intestine, the observed thickening, fusion, and compaction of villi, together with inflammatory cell infiltration in the lamina propria and hyperchromatic epithelial nuclei, indicate chronic mucosal inflammation and epithelial stress [17]. These alterations likely compromise intestinal barrier function, as villous architecture is essential for maintaining absorptive capacity and tight junction integrity [18]. In other vertebrates, similar villus abnormalities are associated with down-regulation of tight junction proteins and increased intestinal permeability, ultimately impairing nutrient absorption and growth [19,20]. The presence of inflammatory infiltration indicates local immune activation [21], which may be associated with the concurrent gut microbiota compositional shifts, but further mechanistic studies are needed to establish a causal link.

In the liver, severe disruption of lobular architecture, hepatocyte vacuolation indicative of lipid accumulation, sinusoidal dilation, and extensive inflammatory infiltration collectively indicate hepatic steatosis with active inflammation [22]. This histopathological pattern is consistent with the elevated hepatosomatic index and lipid-to-body weight ratio, confirming that hepatic lipid accumulation represents a pathological condition rather than a physiological adaptation. The coexistence of steatosis and inflammation is a defining feature of metabolic dysfunction-associated steatotic liver disease (MASLD) in mammals [23], and similar mechanisms may occur in amphibians under metabolic stress [15]. Importantly, hepatic inflammatory infiltration may originate partly from intestinal barrier disruption. Increased intestinal permeability facilitates the translocation of bacterial-derived products, such as lipopolysaccharide, into the portal circulation, thereby triggering or exacerbating hepatic inflammation [24]. These observations are compatible with the concept of gut–liver axis involvement, although they do not directly prove a functional link between intestinal and hepatic abnormalities.

### 4.3. Gut Microbiota Compositional Shifts as a Critical Link Between Phenotype and Internal Mechanisms

Disease often involves alterations in gut microbiota composition, reduced diversity, and impaired function. Conversely, the microbial compositional shifts themselves can disrupt host–microbe interactions, leading to physiological disturbances and subsequent disease [25,26]. Frogs in the MAL group exhibited pronounced gut microbiota compositional shifts, characterized by decreased microbial diversity and modified community structures. Specifically, the relative abundances of Firmicutes were lower, whereas those of Proteobacteria, Fusobacteriota, and Bacteroidota were higher than in controls. A decreased Firmicutes-to-Bacteroidota ratio is a recognized indicator of metabolic dysregulation [27], typically associated with increased dietary energy extraction and adiposity. This aligns well with the observed increases in body weight, lipid-to-body weight ratio, and hepatic steatosis in malformed frogs. Concurrently, the rise in Proteobacteria, a phylum containing numerous opportunistic pathogens, is indicative of gut imbalance and commonly linked to intestinal inflammation and barrier dysfunction [21]. Considering the observed intestinal histopathology (villous fusion and inflammatory infiltration), Proteobacteria enrichment may both contribute to and be intensified by local inflammation, creating a self-sustaining cycle of compromised barrier integrity.

LEfSe analysis further highlighted a distinct differentiation in characteristic microbial taxa between groups, with minimal phylogenetic overlap. This indicates that the malformation syndrome induces systemic restructuring of the gut microbial niche rather than merely altering individual taxa abundances [28]. The CON group displayed enrichment of Actinobacteriota and related lineages, including *Actinobacteria*, *Solirubrobacteraceae*, *Conexibacter*, *Microvirga*, and *Solirubrobacter*. Members of Actinobacteriota have been linked to metabolic regulation and immune homeostasis [29]. In contrast, the MAL group was enriched in predominantly anaerobic fermentative Firmicutes taxa, such as *Peptostreptococcaceae*, *Terrisporobacter*, *Pygmaiobacter*, and *Sporacetigenium*. *Peptostreptococcaceae* members specifically exacerbate intestinal inflammation by activating the NF-κB-NLRP3 pathway and triggering macrophage pyroptosis [30]. Additionally, the enriched Bacteroidota taxa, *Tannerellaceae* and *Macellibacteroides*, possess functional traits associated with inflammation and metabolic dysfunction. *Tannerella forsythia*, representing *Tannerellaceae*, promotes inflammation through methylglyoxal secretion and AGE formation [31] and induces pro-inflammatory cytokine production in host cells [32]. Thus, this dysbiotic microbiome, marked by reduced beneficial Actinobacteriota and increased pro-inflammatory taxa, may contribute to pathogenesis by disrupting metabolic homeostasis and activating systemic inflammation. This pattern is consistent with the systemic phenotypes observed in malformed frogs, including abnormal lipid accumulation, impaired hepatic metabolism, and activated immune responses. These findings indicate that gut microbiota alterations may be associated with both developmental defects and broader metabolic and inflammatory outcomes.

### 4.4. Liver Transcriptome Analysis Reveals Core Mechanisms of Immune Activation and Metabolic Suppression

Given the absence of a reference genome for *P. nigromaculatus*, de novo hepatic transcriptome sequencing was conducted to elucidate the molecular underpinnings of the systemic metabolic disturbances observed in malformed frogs. High-quality assembly and annotation metrics (N50 = 1681 bp; 48.6% annotation rate) established a reliable foundation for downstream analyses. The detection of 2617 DEGs indicates extensive reprogramming of hepatic function, and hierarchical clustering based on these DEGs clearly segregated samples by phenotypic group, confirming that the transcriptional differences are tightly associated with the malformation syndrome.

KEGG pathway analysis, performed separately for up- and down-regulated DEGs, revealed a pronounced transcriptional dichotomy in the livers of malformed frogs, characterized by immune activation paired with broad metabolic suppression. Up-regulated DEGs were significantly enriched in immune-related pathways, including “Neutrophil extracellular trap formation,” “Complement and coagulation cascades,” and “Coronavirus disease–COVID-19.” This enrichment pattern is consistent with sterile inflammation, triggered by endogenous damage-associated molecular patterns (DAMPs) rather than external pathogens [33,34]. In malformed frogs, such DAMPs likely originate from hepatic steatosis, cellular stress, and tissue injury, as supported by histopathological evidence. Although neutrophil extracellular traps (NETs) facilitate the clearance of cellular debris, they can paradoxically exacerbate tissue damage and amplify inflammatory cascades [35]. Similarly, inappropriate or excessive complement activation can intensify liver injury and heighten systemic inflammatory responses [36]. The enrichment of the “Coronavirus disease–COVID-19” pathway, involving genes associated with viral recognition, cytokine signaling, and inflammatory reactions, further underscores the liver’s heightened immune activation, resembling molecular signatures observed in severe viral infections characterized by dysregulated immune responses and endothelial dysfunction [37]. Concurrent up-regulation of the “Ribosome” pathway suggests increased investment in protein synthesis, likely supporting immune and acute-phase responses [38]. Persistent activation of these immune pathways in the liver is associated with chronic inflammation, which has been linked to reduced growth and development in amphibians [39]. This hepatic inflammatory state may be related to growth retardation and malformations, possibly through altered energy allocation.

In stark contrast to this immune activation, down-regulated DEGs revealed a widespread suppression of metabolic pathways. The most significantly suppressed pathways included “Protein digestion and absorption”, “Mineral absorption”, and “Vitamin digestion and absorption”. While these pathways traditionally reflect intestinal functions, their hepatic suppression likely signifies systemic metabolic adjustments to chronic nutrient insufficiency [40]. Given the reduced intestinal length and villus abnormalities observed in malformed frogs, compromised nutrient absorption at the intestinal level may lead to systemic nutrient deprivation, prompting the liver to down-regulate genes involved in nutrient processing when substrates are limited [41]. Critical lipid metabolism pathways, including “Linoleic acid metabolism” and “Ether lipid metabolism”, were also significantly down-regulated [42]. This finding corresponds to the observed metabolic phenotype featuring elevated lipid-to-body weight ratios and hepatic steatosis, suggesting that, despite excessive lipid accumulation, the hepatic capacity for lipid oxidation and utilization is reduced, a paradox indicative of dysfunctional lipid handling rather than efficient lipid storage.

In-depth analysis of the PPAR signaling pathway and the renin–angiotensin system (RAS) further pinpointed core nodes of metabolic disruption. Within the PPAR pathway, key genes involved in fatty acid uptake, activation, and β-oxidation, including *FABP1*, *CD36*, *ACSL1*, and *CPT1B*, were significantly down-regulated. This transcriptional profile indicates a severe impairment in hepatic fatty acid oxidation, which likely contributes substantially to the intrahepatic and systemic lipid accumulation observed in malformed frogs [43,44,45,46]. Simultaneously, all detected differentially expressed genes in the RAS pathway, *ACE*, *ACE2*, *CPA3*, *AGTR1*, *ENPEP*, and *ANPEP*, were down-regulated. The RAS plays established roles in organ development, angiogenesis, and nutrient delivery in vertebrates [47]. The widespread down-regulation of RAS-associated genes observed in malformed frogs is noteworthy and may reflect either persistent transcriptional alterations resulting from early developmental disturbances or an adaptive response to ongoing metabolic and inflammatory stress [48]. Regardless of the cause, simultaneous suppression of both the PPAR and RAS pathways indicates significant disruption in metabolic regulation and early developmental processes, which may be associated with the complex phenotypic features of the syndrome.

### 4.5. Aquaculture Implications and Future Research Directions

From an aquaculture standpoint, the findings presented in this study have multiple practical implications. First, the observed disruptions in the gut–liver axis raise the possibility that monitoring of intestinal health and hepatic function may serve as early indicators for malformation risk in farmed frog populations. Second, the association identified between gut microbiota alterations and systemic pathology suggests that probiotic interventions could be explored as a potential strategy to decrease malformation incidence. Third, the identified metabolic disruptions indicate that optimizing nutritional regimens may be important for preventing developmental abnormalities. Future research should investigate whether targeted modulation of the gut microbiome or host metabolic pathways can effectively mitigate malformation rates in commercial aquaculture environments.

## 5. Conclusions

This study reveals that the “short-leg” malformation syndrome in *P. nigromaculatus* is associated with systemic alterations extending beyond limb abnormalities, including gut microbiota compositional shifts, concurrent histopathological damage in the intestine and liver, and hepatic transcriptional reprogramming characterized by immune activation and metabolic suppression. Together, these findings provide a novel systemic perspective beyond localized limb defects and establish a multi-dimensional framework for future intervention strategies in frog aquaculture.

## Figures and Tables

**Figure 1:**
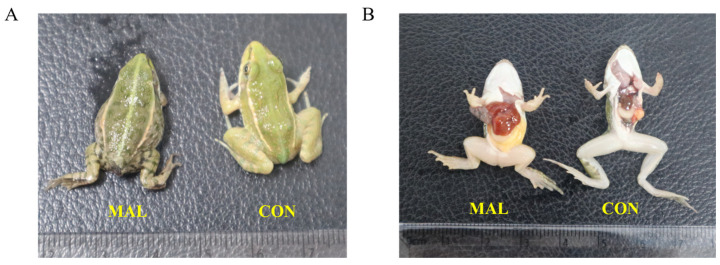
Comparative external morphology of post-metamorphic frogs between control and malformed groups. (**A**) Dorsal view showing differences in body size and coloration between the CON and MAL groups. (**B**) Ventral view illustrating differences in visceral contours and limb development.

**Figure 2:**
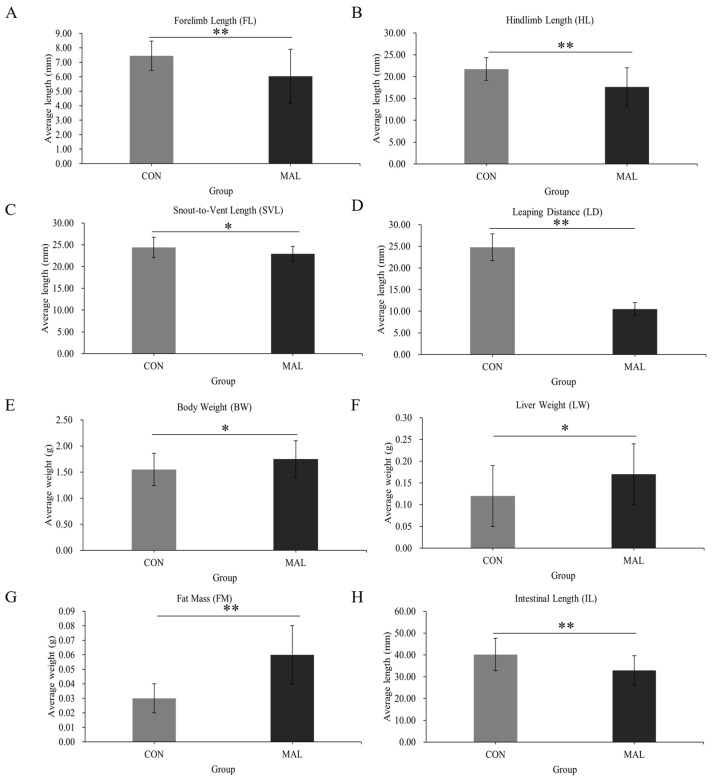
The basic growth performance parameters of each group. (**A**) FL, forelimb length; (**B**) HL, hindlimb length; (**C**) SVL, snout-to-vent length; (**D**) LD, leaping performance; (**E**) BW, body weight; (**F**) LW, liver weight; (**G**) FM, fat mass; (**H**) IL, intestinal length. * *p* < 0.05, ** *p* < 0.01.

**Figure 3:**
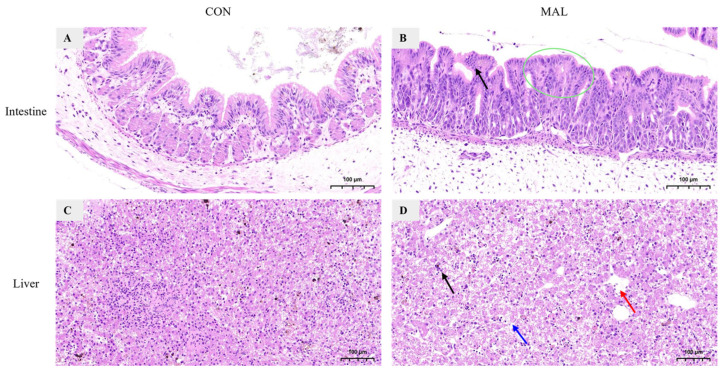
Histopathological alterations in intestinal and liver tissues of malformed *P. nigromaculatus* revealed by H&E staining. (**A**,**B**) Representative intestinal sections (scale bars = 100 μm). (**C**,**D**) Representative liver sections (scale bars = 100 μm). Green circles indicate partially fused intestinal villi with inflammatory cell infiltration; black arrows indicate inflammatory cell infiltration; red arrows indicate vacuolation; blue arrows indicate hepatic sinusoid dilation.

**Figure 4:**
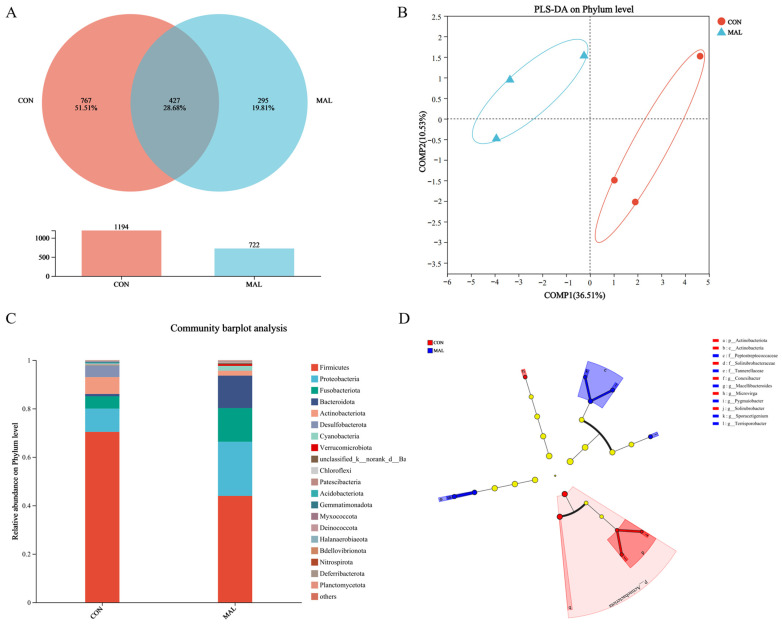
Comparative analysis of gut microbiota between CON and MAL *P. nigromaculatus*. (**A**) Venn diagram showing shared and unique OTUs. (**B**) Principal coordinates analysis (PCoA) based on Bray–Curtis distance, illustrating microbial community separation. (**C**) Relative abundance of bacterial taxa at the phylum level. (**D**) LEfSe-derived LDA score histogram identifying differentially abundant taxa (LDA score > 2.0).

**Figure 5:**
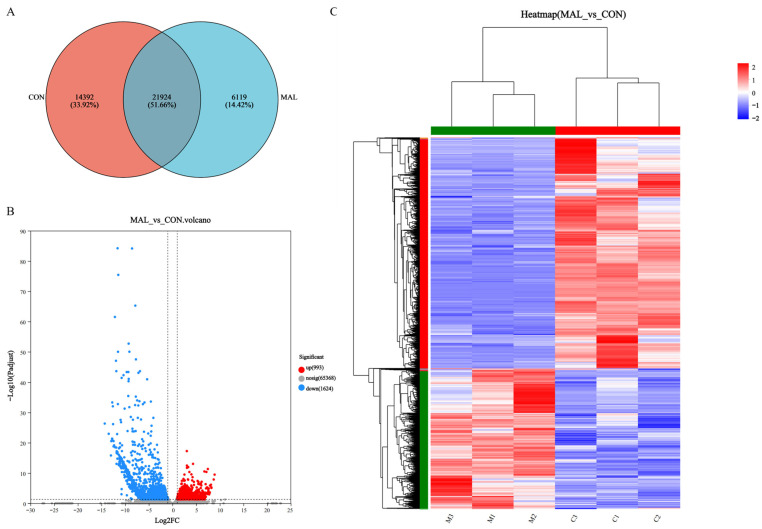
Global analysis of hepatic DEGs in malformed frogs. (**A**) Venn diagram showing expressed genes shared between CON and MAL groups. (**B**) Volcano plot of DEGs. Red dots, up-regulated genes; blue dots, down-regulated genes; gray dots, non-significant genes (|log_2_FC| > 1, FDR < 0.05). (**C**) Hierarchical clustering heatmap of all DEGs. Rows represent genes, and columns represent samples. Blue to red indicates low-to-high normalized expression, clearly separating the CON and MAL groups.

**Figure 6:**
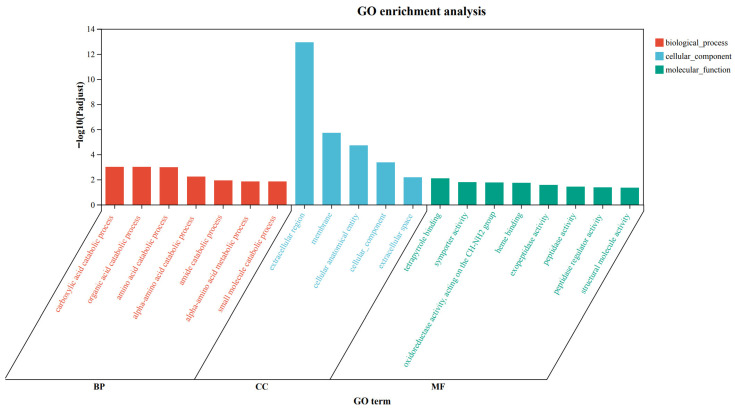
GO enrichment analysis of hepatic DEGs in malformed *P. nigromaculatus*. The top 21 significantly enriched GO terms (padjust < 0.05) are shown and classified into BP, CC, and MF. Bar length represents –log10(padjust).

**Figure 7:**
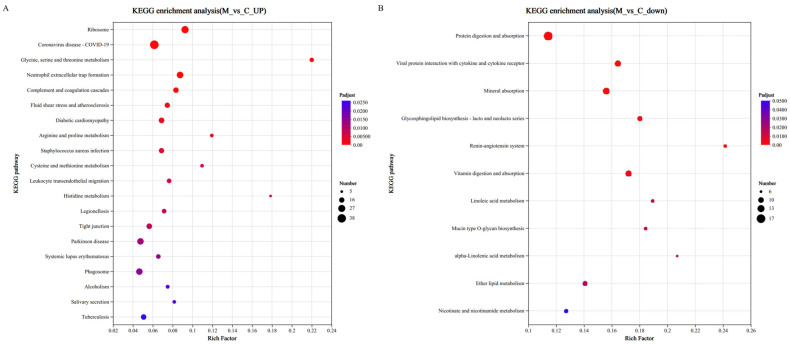
KEGG pathway enrichment analysis of hepatic DEGs in malformed *P. nigromaculatus*. Enrichment results are shown for up-regulated (**A**) and down-regulated (**B**) genes (padjust < 0.05). The rich factor indicates enrichment degree, point size represents DEG count, and color corresponds to –log10(padjust).

**Figure 8:**
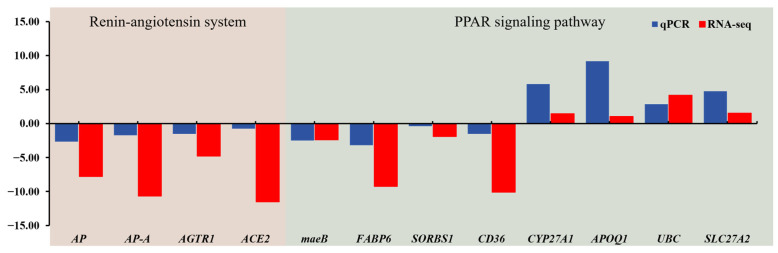
Comparison of RNA-seq and qPCR results. Gene expression was normalized to 18S rRNA. Red bars, RNA-seq; blue bars, qPCR. log_2_ fold change > 0 indicates higher expression in MAL, whereas log_2_ fold change < 0 indicates higher expression in CON.

**Table 1:** Statistical summary of transcriptome sequencing data.

Sample Name	Clean Reads	Q20 (%)	GC Content (%)	Total Mapped Reads	Uniquely Mapping Rate (%)
CON_1	20,136,479	95.74	43.70	16,589,529	82.39
CON_2	19,912,461	95.87	43.81	16,765,524	84.20
CON_3	20,207,686	95.87	44.11	16,926,904	83.76
MAL_1	21,920,303	95.89	44.30	18,653,798	85.10
MAL_2	22,056,240	95.78	44.15	18,633,291	84.48
MAL_3	22,861,489	95.81	44.10	19,362,431	84.69

**Table 2:** DEGs involved in the PPAR signaling and renin–angiotensin pathways in malformed frogs compared to controls.

KO ID	Gene Name	log2 (Fold Change) (MAL vs. CON)	Adjusted *p* Value
PPAR signaling pathway			
*K06259*	*CD36*	−10.123	5.38 × 10^−32^
*K08750*	*FABP1*	−5.666	0.049
*K19523*	*CPT1B*	−3.677	0.004
*K00029*	*meaB*	−2.404	4.32 × 10^−10^
*K01897*	*ACSL1*	−1.832	0.008
*K06086*	*SORBS1*	−1.917	0.01
*K08770*	*UBC*	4.159	0.017
*K01596*	*E4.1.1.32*	1.391	0.002
Renin-angiotensin system			
*K09708*	*ACE2*	−11.537	1.76 × 10^−33^
*K11141*	*ENPEP*	−7.798	3.73 × 10^−7^
*K04166*	*AGTR1*	−4.777	0.002
*K11140*	*ANPEP*	−4.610	5.06 × 10^−23^
*K08780*	*CPA3*	−3.939	0.001
*K01283*	*ACE*	−3.759	5.61 × 10^−11^

## Data Availability

The sequencing data generated in this study have been deposited in the NCBI Sequence Read Archive (SRA) under BioProject ID PRJNA1473097. The data are publicly available as of the date of publication.

## Supplementary Materials

The following supporting information can be downloaded at: https://www.mdpi.com/article/10.3390/ani16132069/s1, Figure S1: Characterization of the de novo liver transcriptome of *P. nigromaculatus*. (A) Unigene length distribution; (B) Summary of functional annotations of unigenes and transcripts; Figure S2: Disruption of key signaling pathways in the liver of malformed *P. nigromaculatus*. (A) PPAR signaling pathway. (B) Renin-angiotensin system. Blue, significantly down-regulated genes; red, significantly up-regulated genes in the MAL group; Table S1: Primer sequences used for transcriptome validation; Table S2: Comparison of key physiological ratios between CON and MAL *P. nigromaculatus*; Table S3: List of DEGs in MAL vs. CON. Table S4: GO terms enriched in MAL vs. CON; Table S5: KEGG pathways enriched in MAL vs. CON.
