# Supplementary figures and images for: Multi-Omics Reveals Gut Microbiota Shifts and Hepatic Metabolic–Immune Alterations in “Short-Leg” Malformed Frog (Pelophylax nigromaculatus)
Source: Animals (Basel). 2026 Jul 4;16(13):2069. doi: 10.3390/ani16132069 (PMC13359810; doi:10.3390/ani16132069)

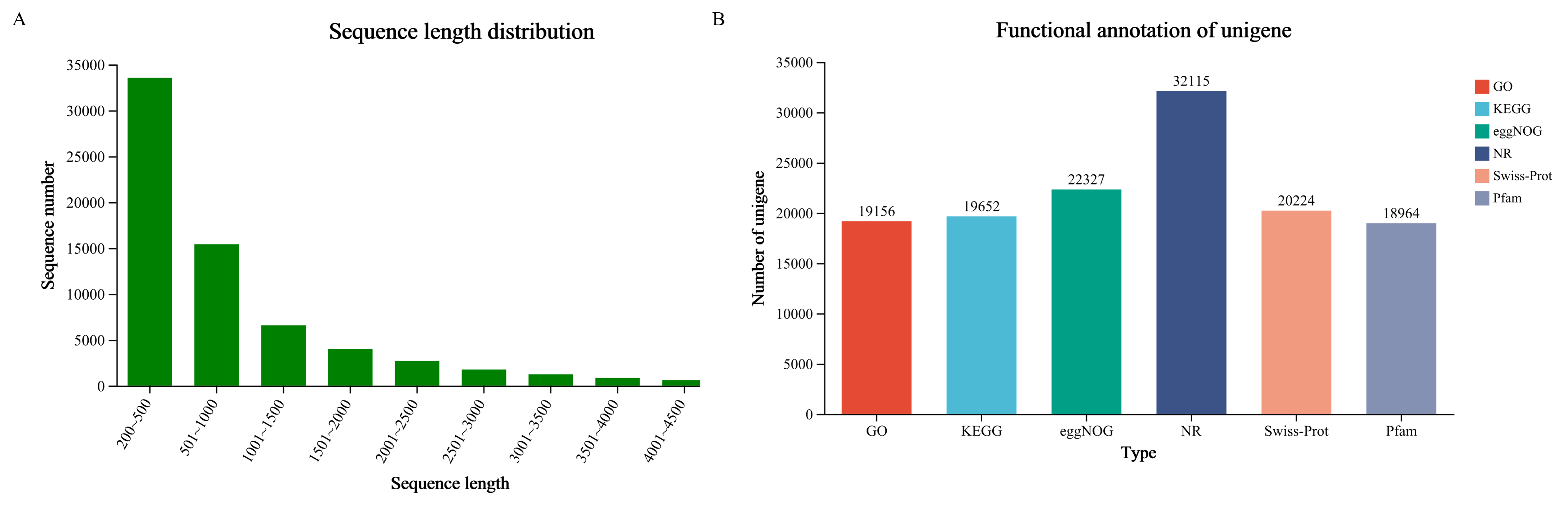

Supplement: Supplementary file 1 [file animals-16-02069-s001.zip › Figure S1.tif]

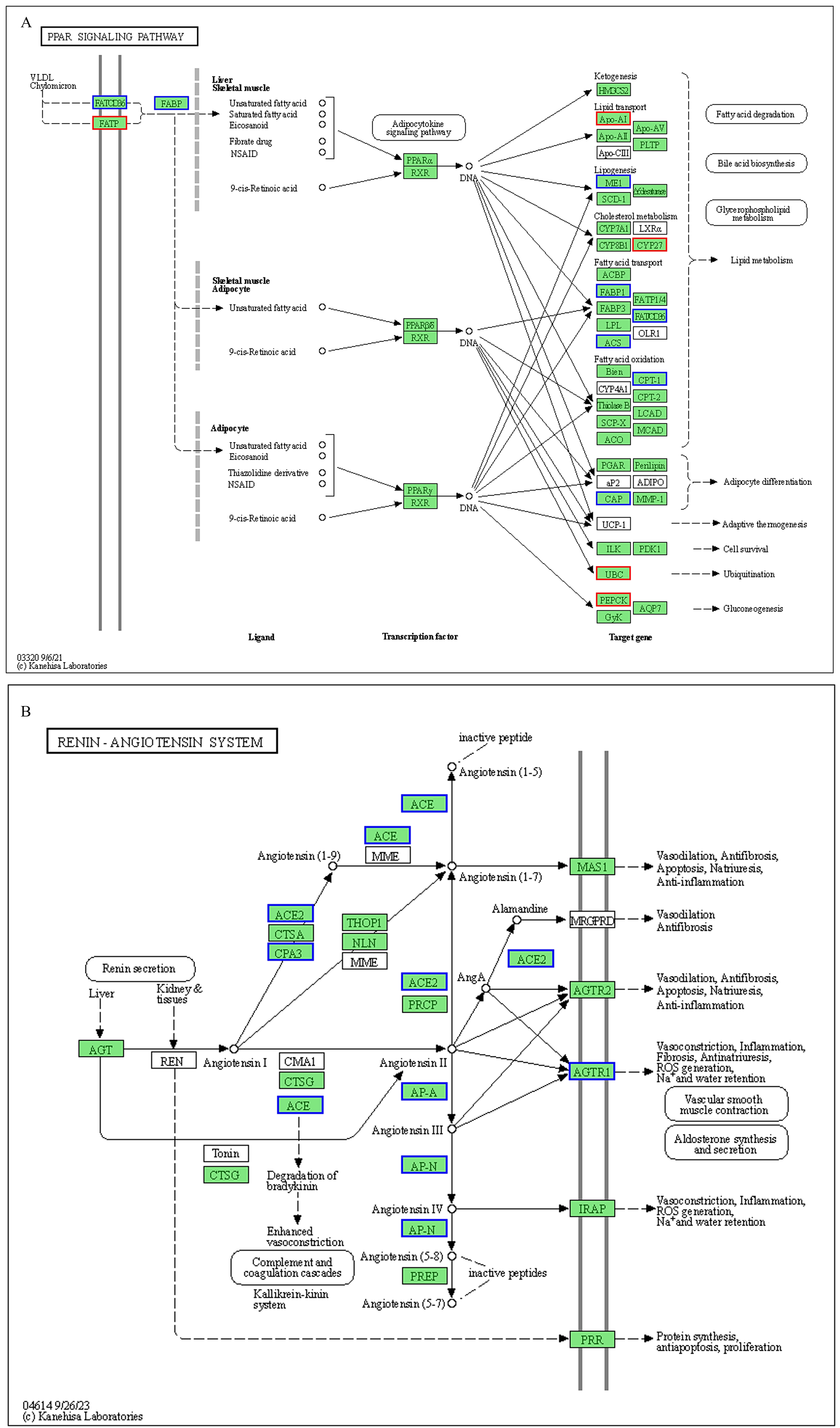

Supplement: Supplementary file 1 [file animals-16-02069-s001.zip › Figure S2.tif]
